# Interrelation Between Fibroblasts and T Cells in Fibrosing Interstitial Lung Diseases

**DOI:** 10.3389/fimmu.2021.747335

**Published:** 2021-11-05

**Authors:** Yunxin Lai, Xinru Wei, Ting Ye, Lilin Hang, Ling Mou, Jin Su

**Affiliations:** ^1^State Key Laboratory of Respiratory Disease, National Clinical Research Center for Respiratory Disease, Guangzhou Institute of Respiratory Health, The First Affiliated Hospital of Guangzhou Medical University, Guangzhou, China; ^2^Zhujiang Hospital of Southern Medical University, Guangzhou, China

**Keywords:** fibroblasts, T cells, interrelation, ILDs, fibrosis

## Abstract

Interstitial lung diseases (ILDs) are a heterogeneous group of diseases characterized by varying degrees of inflammation and fibrosis of the pulmonary interstitium. The interrelations between multiple immune cells and stromal cells participate in the pathogenesis of ILDs. While fibroblasts contribute to the development of ILDs through secreting extracellular matrix and proinflammatory cytokines upon activation, T cells are major mediators of adaptive immunity, as well as inflammation and autoimmune tissue destruction in the lung of ILDs patients. Fibroblasts play important roles in modulating T cell recruitment, differentiation and function and conversely, T cells can balance fibrotic sequelae with protective immunity in the lung. A more precise understanding of the interrelation between fibroblasts and T cells will enable a better future therapeutic design by targeting this interrelationship. Here we highlight recent work on the interactions between fibroblasts and T cells in ILDs, and consider the implications of these interactions in the future development of therapies for ILDs.

## Introduction

The interstitial lung diseases (ILDs) are a large, heterogeneous group of several hundred generally rare pulmonary pathologies, involving injury, inflammation and/or scarring in the lung. Fibrosing ILDs, especially idiopathic pulmonary fibrosis (IPF) with unknown aetiology, manifest a progressive phenotype characterized by decline in lung function, life quality and early mortality. To date, only two approved drugs (Nintedanib and Pirfenidone) are available for IPF, which can only delay disease progression. Connective tissue diseases (CTDs) are a heterogeneous group of autoimmune disorders that can affect any of the body’s connective tissues, which frequently evolve to ILDs (so called CTD-ILDs) (1). Rheumatoid arthritis-associated ILDs (RA-ILDs)and systemic sclerosis-associated ILDs (SSc-ILDs) are two major CTD-ILDs. As many as 10% patients with RA have been diagnosed with ILDs over the course of the disease (2). Both genetic and environmental factors are involved in the development of ILDs. The mutations of genes critical in telomere maintenance are well-known for their involvement in IPF. Exposure to air pollutants such as ozone (O3), nitrogen dioxide (NO2) and particulate matter may trigger oxidative stress and chronic inflammation (3), which accelerate telomere shortening and dysfunction, and critically short telomeres trigger genomic instability and cellular senescence (4), contributing to the development of ILDs. In addition, respiratory infections are increasingly deemed as critical causes in ILDs pathogenesis.

The lung contains more than 40 different cell types and cellular interactions between them are extremely complex which take part in the pathogenesis of ILDs (5). Fibroblasts have been acknowledged as a ‘non-classical’ branch of the innate immunity (6). It is suggested that chronic inflammation occurs because of disordered fibroblast behavior in which failure to switch off their inflammatory program leads to the inappropriate survival and retention of leukocytes within inflamed tissue (7). Accordingly, fibroblasts are important sentinel cells that play a critical role in the switch from acute inflammation to adaptive immunity and tissue repair. T cells are the most important inducers of adaptive immunity which clear infections and also cause autoimmunity under specific conditions. Whether T cells are necessary for the development of ILDs remains unknown. Although bleomycin causes lung fibrosis in immunodeficient mice without T cells (8), bleomycin based murine models cannot truly recapitulate the characteristics of IPF in humans. T cell anomaly may cause repetitive alveolar epithelial injury and repair which lead to gradual destruction of functional lung parenchyma and its replacement by extracellular matrix (ECM). Furthermore, failure to remove excessive numbers of apoptotic cells may induce a persistent inflammatory state. Global Treg impairment has been found in IPF patients which strongly correlates with disease severity (9). While the fibroblasts-macrophages reciprocal interactions in ILDs have gained much attention (10), the interactions between fibroblasts and T cells in ILDs are underrated. In this review, we will summarize recent research advances on the roles of fibroblasts and T cells and the interrelation between them in infections and ILDs, in hope to ignite new interests in this area.

## Plastic Fibroblasts are Drivers of ILDs

**Figure 1:**
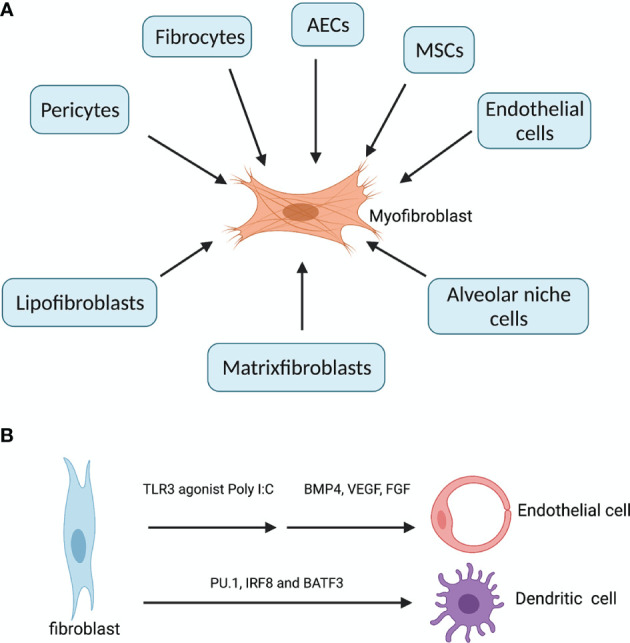
Diverse origins of myofibroblasts and transdifferentation of fibroblast. **(A)** In fibrosing ILDs, the effector myofibroblasts can be derived from three other resident fibroblasts (lipofibroblasts, matrixfibroblasts and alveolar niche cells), pericytes (residing within basal membranes or perivascular linings), hematopoietic CXCR4^+^ fibrocytes, alveolar epithelial cells (AECs), endothelial cells (ECs) and mesenchymal stem cells (MSCs) (14). **(B)** fibroblasts can transdifferentiate into endothelial cells when treated with innate immune agonist Poly I:C and growth factors BMP4, VEGF and FGF. Fibroblasts overexpressing PU.1, IRF8 and BATF3 simultaneously can be reprogrammed into functional dendritic cells. Created with BioRender.com.

Fibroblasts are mesenchymal cells residing in all tissue types. The primary function of fibroblasts is the maintenance of the structural integrity of the connective tissues, through the secretion of ECM proteins such as collagens, matrix metalloproteinases (MMPs) and tissue inhibitors of metalloproteinases (TIMPs). However, aberrant accumulation of activated fibroblasts (myofibroblasts) and ECM may lead to organ fibrosis, a hallmark of ILDs. Lung myofibroblasts in injury and fibrosis are heterogenous in terms of their origins. The predominant sources are pericytes (11) and resident fibroblasts, along with minor sources such as hematopoietic CXCR4^+^ fibrocytes (12), alveolar epithelial cells (AECs) (13), endothelial cells and MSCs (14) (**Figure 1A**). Foxd1 progenitor–derived lung pericytes, localized within basal membranes or perivascular linings, are a major source of myofibroblasts after lung injury (11), as well as in kidney fibrosis (15). Increased number of circulating fibrocytes was detected in subjects with autoimmune ILDs compared with healthy controls (16). AECs trans-differentiate into fibroblasts through epithelial-mesenchymal transition (EMT), a process requiring prolonged exposure to TGFβ for nearly 2 weeks, and AECs overlying fibroblastic foci in IPF/UIP appear histologically similar to fibroblasts, suggesting ongoing EMT (13). Recently, a unique HAS1^hi^ ECM-producing fibroblast subset was identified markedly enriched in lungs from patients with IPF, and a population of epithelial cells (KRT5^-^/KRT17^+^) expressing collagen and other ECM components was found conserved across a subset of histopathologic patterns of pulmonary fibrosis (17). Interestingly, human fibroblasts can transdifferentiate to endothelial cells (ECs) through TLR3 (toll-like receptor 3) agonist Poly I:C induced innate immune signaling and ECs growth factors (18). In addition, fibroblasts can be reprogrammed into functional antigen-presenting DCs when overexpressing three transcription factors PU.1, IRF8 and BATF3 (19) (**Figure 1B**). Therefore, fibroblasts are not only effectors of fibrosis, but also hold the potential to reorganize tissue infrastructures through ECM and transdifferentiation.

Alveolar resident fibroblasts comprise four functionally distinct populations: myofibroblasts, lipofibroblasts, matrixfibroblasts and alveolar niche cells. Lipofibroblasts, matrixfibroblasts and alveolar niche cells can transdifferentiate into myofibroblasts, and the imbalance between these populations are associated with various fibrotic ILDs (20). While myofibroblasts are driver of fibrosis, matrixfibroblasts, lipofibroblasts and alveolar niche cells are regulators of alveolar epithelial cell growth and differentiation from type II to type I cells. In IPF, the proliferating myofibroblasts within the fibroblastic foci are Thy-1^-^, whereas normal lung fibroblasts are predominantly Thy-1^+^, and Thy-1^-^ myofibroblasts are more resistant to apoptosis than Thy-1^+^ ones (21). The resistance to apoptosis by lung fibroblasts from IPF patients has been well established. IPF lung fibroblasts are resistant to Fas-mediated apoptosis, probably through anti-apoptotic proteins ILP and FLIP_L_ (22). Aged lung fibroblasts from IPF patients show persistent activation of mTOR and reduced autophagy activity, which contributes to apoptosis resistance (23). Moreover, mitochondrial dysfunctions such as inhibited opening of mitochondrial permeability transition pore (mPTP) and reduced release of cytochrome c contribute to apoptosis resistance by IPF fibroblasts (24). FasL^+^ myofibroblasts from fibrotic lungs can induce lung epithelial cell apoptosis through Fas ligation in recipient mice (25). Normally fibroblasts are early players in initiating irritation in response to invading microorganisms and tissue damage; they respond to wound healing through proliferating and migrating to the sites of tissue injury to restructure the ECMs; and they also monitor any deviation from tissue homeostasis by sensing changes in mechanical stress through integrin connectors which substantially link ECM with their cytoskeleton, enabling transmission of force in both directions. Therefore, the plastic and versatile fibroblasts are heterogenous in terms of both origins and functions. Blocking the profibrotic programs in fibroblasts is key to the treatment of various fibrosing ILDs. It has been shown that simply eliminating the activated fibroblasts through FAP-specific chimeric antigen receptor T cells showed therapeutic efficacy against fibrosis (26).

## Persistent Infections Are Associated With ILDs Development

Lung microbiome is deemed increasingly as a notable player in the initiation and exacerbation of ILDs. Humans are in constant combat with respiratory viruses such as influenza virus, coronavirus, Epstein-Barr virus (27) and adenovirus (28), and bacteria such as *mycobacterium tuberculosis* and *Haemophilus influenzae*, all of which may play key roles in the pathogenesis of ILDs (29). Defects in the immune system, when dealing with lung microbiome, may be key drivers for ILDs development. Mutation of the innate sensor TLR3, which recognizes viral dsRNA, is associated with IPF and pulmonary sarcoidosis (30, 31). Staphylococcus nepalensis released corisin, a peptide conserved in diverse staphylococci, induces apoptosis of lung epithelial cells and is upregulated in IPF patients with acute exacerbation compared to patients without disease exacerbation (32). Microbial DNA with hypomethylated CpG motifs, ligand for innate immune receptor TLR9, promotes profibrotic cytokine and chemokine synthesis in IPF fibroblasts which is associated with the rapidly progressive IPF phenotype (33). Innate immune receptor TLR4 deficiency was shown to exacerbate pulmonary fibrosis through promoting formation of an immunosuppressive tissue microenvironment and attenuating autophagy-associated degradation of collagen (34). A substantial proportion of patients with acute respiratory distress syndrome (ARDS) following viral infection developed or even died from progressive pulmonary fibrosis (35), and the rate of fibrosis was positively correlated with disease duration (36). Influenza infection in aged mice leads to non-resolving inflammation and persistent chronic lung pathology, and age-associated lung CD8 T_RM_ accumulation is not protective but rather drives inflammatory and fibrotic sequelae after primary respiratory viral infection (37). These studies indicate that failure to eradicating infectious agents from the lung can be a primary cause to ILDs development.

## Lung T_RM_ Cells Balance Fibrotic Sequelae With Protective Immunity

**Figure 2:**
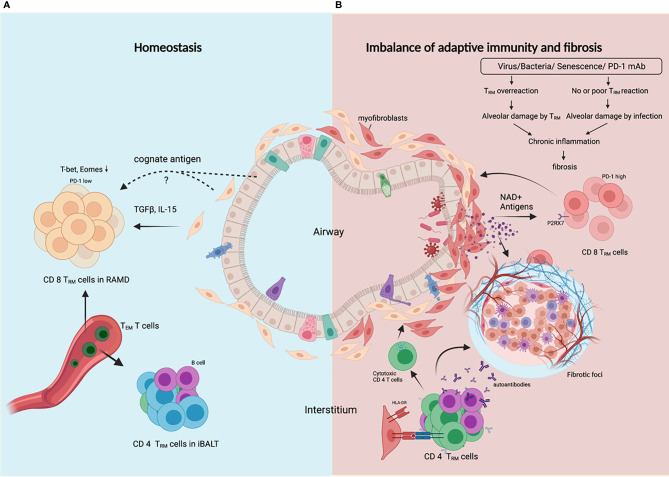
T_RM_ cells control the balance between protective adaptive immunity and progressive fibrosis. **(A)** In homeostasis following resolution of lung infection or injury, CD8 T_RM_ cells and CD4 T_RM_ cells are generated and transiently maintained in RAMD and iBALT respectively, and can be replenished by T_EM_ cells from the bloodstream. T_RM_ cells are poised to render robust protection against secondary infection or injury, and ensure subsequent rapid tissue repair. **(B)** In fibrosing lung, dysregulated CD8 T_RM_ cells in response to certain external insults lead to constant alveolar damage, subsequent chronic inflammation and progressive fibrosis. Alveolar damage release high level of NAD^+^ which selectively delete P2RX7^+^ T_RM_ cells. CD4 T_RM_ cells in iBALT may help B cells generate autoantibodies, or become cytotoxic or regulatory CD4 T cells that worsen tissue injury and fibrosis. Created with BioRender.com.

Resident memory T (T_RM_) cells take up residence in the lung after respiratory infections to facilitate rapid and localized immune responses during reinfection. Lung T_RM_ cell pool comprises two distinct subsets, airway T_RM_ cells and interstitial T_RM_ cells, the homeostasis of which are regulated by integrated stress response (ISR) induced by airway cues such as viral infection and amino acid starvation (38). CD8 T_RM_ cells typically localize in the epithelial layers of barrier tissues where they are optimally positioned to act as sentinels to trigger antigen-specific protection, while CD4 T_RM_ cells typically localize below the epithelial layers and cluster in lymphoid structures designed to optimize interactions with antigen-presenting cells (39). However, in the lung, CD4 T_RM_ cells in the lung interstitium are maintained predominantly within inducible bronchus-associated lymphoid tissue (iBALT), and interstitial CD8 T_RM_ cells are predominantly maintained within the repair-associated memory depots (RAMD) that are temporarily created at the site of tissue injury which is crucial for protection against secondary infections (40, 41). The structural characteristics of RAMD differ from iBALT as most CD8 T_RM_ cells in the RAMD do not form organized lymphoid structures. The size of the RAMD shrinks over time as tissue repair proceeds and tends to disappear several months post-infection. TGFβ signaling is required for T_RM_ cell development in peripheral tissues, and IL-15 signaling is required for the maintenance of CD8 T_RM_ cells. Coordinated downregulation of both T-bet and Eomes in CD8^+^ T_RM_ cells was shown to be required for optimal TGFβ signaling and residual level of T-bet was necessary for IL-15R (CD122) expression (42). Moreover, long-term T_RM_ cell maintenance depends on local persistence of cognate antigens (43), but it is still unresolved which type of cells these antigens persist in (**Figure 2A**). It is interesting to speculate that lung resident fibroblasts are ideal hosts for these antigens since they are resistant to external stress and persist long.

Lung T_RM_ cells express high level of PD-1, a marker reflecting tissue residency rather than exhaustion (44). Peripheral T cells in age-related IPF and sarcoidosis also showed upregulated PD-1, reduced proliferative capacity and increased TGFβ and IL-17A production (45). T_RM_ cells have been demonstrated to promote chronic parenchymal inflammation and fibrosis in aged mice following viral infection, and aged T_RM_ cells are insufficient to provide protective immunity due to defects in genes involved in TCR signaling and effector function (37). For PD-1^+^ Th17 cells, PD-1 blockade is antifibrotic for reducing IL-17A and TGFβ expression, but for PD-1^hi^ T_RM_ CD8 T cells, PD-1 blockade is profibrotic for disrupting the balance between protective immunity and fibrotic sequelae controlled by T_RM_ cell ‘exhaustion’ (46). In light of this, lung fibrosis caused by PD-1 blockade therapies in lung cancer patients can be attributed to activation of unrelated viral T_RM_ cells. Unlike T cells in lymphoid organs, lung T_RM_ cells can be reactivated by not only CD11c^+^ DCs, but also non-hematopoietic cells (47), and excessive bystander activation of lung T_RM_ cells may result in amplified inflammatory and fibrotic signals contributing to the development and/or exacerbation of preexisting fibrotic respiratory diseases.

Alveolar damage is a hallmark of progressive ILDs, which induces senescence of fibroblasts and depletion of T_RM_ cells. External insults like irradiation cause DNA damage and DNA damage response (DDR) in fibroblasts. GMP-AMP synthase (cGAS) is crucial in perpetuating IPF lung fibroblast senescence by binding damaged DNA released into the cytosol (12). Lung fibroblasts upregulate type I interferon in response to microbial or self DNA (48). Local concentration of extracellular ATP and NAD^+^ is strongly increased during tissue damage and inflammation, as both nucleotides are released into the extracellular space. Their receptor, P2RX7, is expressed on T_RM_ cells and P2RX7 activation by NAD^+^ resulted in selective cell death of _TRM_ cells (49) (**Figure 2B**). However, P2RX7 sensing of ATP promotes CD8 T_RM_ cell generation by enhancing their sensitivity to TGFβ (50), perhaps a dominant effect in healthy lungs without alveolar damage. The gradual shrinkage of lung T_RM_ cell pool caused by destruction of alveoli and fibrosis may hamper immunity against recurrent infections and allow for further inflammation and fibrosis.

## Autoreactive and/or Cytotoxic CD4 T Cells Drive the Development of ILDs

Cellular autoreactivities are integral players in idiopathic pulmonary fibrosis (51). Certain autoantigens have been identified, such as nuclear factor (52) annexin 1 (53), alanyl-tRNA synthetase (54) and HSP-70 (55), which also generate antibody responses presumably with the help ofCD4 T cells with the same antigen-specificity. Epstein–Barr protein has homology to alanyl-tRNA synthetase, which suggests a link between viral infection and autoimmunity in ILDs. HLA-DR alleles are involved in the pathogenesis of ILDs. DRB1*1501 is over-represented in IPF patients (56, 57). A self-epitope has been identified from the α3 chain of Type IV collagen (α3_135-145_). HLA-DR15-α3_135-145_ recognizing CD4 T cells exhibit a conventional T cell phenotype that secretes pro-inflammatory cytokines; however, HLA-DR1-α3_135-145_ recognizing T cells are predominantly Tregs expressing tolerogenic cytokines (58). Only recently, widespread HLA-DR expression on lung epithelial and endothelial cells was found in COVID-19 patients, accompanied by increased cytotoxic CD4 T cells in lung infiltrate, contributing to increased apoptosis of epithelial cells, lung inflammation and eventually to fibrosis in severe COVID-19 (59). Moreover, abnormal CD4 T cell clonal expansions were found in all IPF patients, with 82% of these subjects also generating IgG autoantibodies against cellular antigens. These CD4 T cells have characteristics typical of cell-mediated pathologic responses, including augmented effector functions, help for autoantibody production (51). Alveolar CD103^+^ resident CD4 T cells from the bronchoalveolar lavage fluid of ILDs patients were shown to exert a Th1-like inflammatory phenotype (60), and these cells may play a notorious role in the aggressive injury of alveoli. Human lung fibroblasts have been proved to engulf live nontypeable *Haemophilus influenzae* (NTHi), bacteria highly prevalent in human respiratory tract, and present antigens to CD4 T cells through HLA-DR, inducing IFNγ and IL-17A production (61). These studies suggest that lung fibroblasts might play a crucial role in regulating the balance between inflammatory, cytotoxic and regulatory CD4 T cells and autoreactive and/or cytotoxic CD4 T cells may be critical contributors or even drivers in the pathogenesis of ILDs (**Figure 2B**).

## Interrelation of Fibroblasts and T Cells in the Formation of iBALT

Tertiary lymphoid structures (TLS) are present in tumors, infected and inflamed tissues including the lung with ILDs. TLS formation begin with an initial phase of stromal cell priming that occurs independently of lymphotoxin and precedes tissue infiltration by adaptive immune cells, followed by the maturation of fibroblasts to a full lymphoid tissue organizer cell phenotype which appears to be dependent in most settings on lymphotoxin and TNFα (62, 63). Transient activation of stromal cells that often occurs in acute phases of inflammation is not sufficient to support complete lymphoid-like fibroblast maturation. Upon resolution of inflammation, the “primed state” of fibroblasts is likely to be lost, but antigen persistence or chronic inflammation may drive the development of lymphoid tissue-like mesenchyme.

In respiratory infections, iBALT as one type of TLS, serves as a general priming site for T cells (64), and dissolve upon antigen clearance (64). The role of fibroblasts in TLS formation and function has been extensively elaborated elsewhere (62, 63). Viral infection induced type I IFN upregulates CXCL13 in pulmonary PDGFRα^+^ fibroblasts which recruit CXCR5^+^ B cells to support ectopic germinal center formation (65). Resident CD4^+^ T cells tightly colocalize with B cells in iBALT and promote humoral responses against viral infection in the lung (66). IL-17 produced by γδ T cells has been demonstrated to provide the trigger for priming of lung fibroblasts in iBALT formation (67, 68). And Th2-derived IL-13 and Th17-derived IL-17A synergistically stimulate pulmonary fibroblasts to produce CXCL13 which is required for iBALT formation (69). Autoantibodies generated in iBALT may further worsen fibrosis, which is exemplified by antifibroblast autoantibodies capable of binding to the surface of fibroblasts and induce profibrotic chemokines (70).

## Interrelation Between Fibroblasts and T Cells in Inflammation and Fibrosis

### Contact-Dependent Crosstalk Between Fibroblasts and T Cells

Fibroblasts and T cells interact with each other in cell contact-dependent manners in all tissues, healthy, inflamed, fibrotic, or tumors. This interaction regulates tissue remodeling and immune responses. Activated T cells inhibit collagen I and III production by dermal and synovial fibroblasts (71, 72), but stimulate FLS cells to produce proinflammatory cytokines in a contact-dependent manner which required membrane-bound TNFα, but not LFA-1/ICAM-1 (73). Coculture of T cells with bronchial fibroblasts, which involves CD40L/α5β1 interaction, increases the production of IL-6 by fibroblasts (74). T-cells overexpressing the integrins αvβ3 and αvβ5 are profibrotic on cultured primary human pulmonary fibroblasts, probably through a TGFβ-dependent mechanism (75). The contact between synovial fibroblasts and T cells induces ICAM-1 and VCAM-I expression in fibroblasts, and TNFα, IFNγ and IL-6 secretion from T cells; however, contact between dermal fibroblasts and T cells only induces ICAM-1 on fibroblasts, suggesting tissue-dependent outcome (76). Another study showed that coculture of either Th1 or Th17 cells with synovial fibroblasts promoted CD40、CD54 and MHC-II expression and production of IL-6 and IL-8 by synovial fibroblasts (77). Normal fibrocytes contact with naive CD4 T cells *in vitro* induced release of Th2 cytokines IL-4 and IL-10, but trans-differentiation of fibrocytes reversed Th2 cytokine production (78). Th17 cells promote inflammatory and antigen-presenting functions of fibrocytes in autoimmunity (79). Fibrocytes were also shown to be potent stimulators of anti-virus cytotoxic T cells (80).

**Figure 3:**
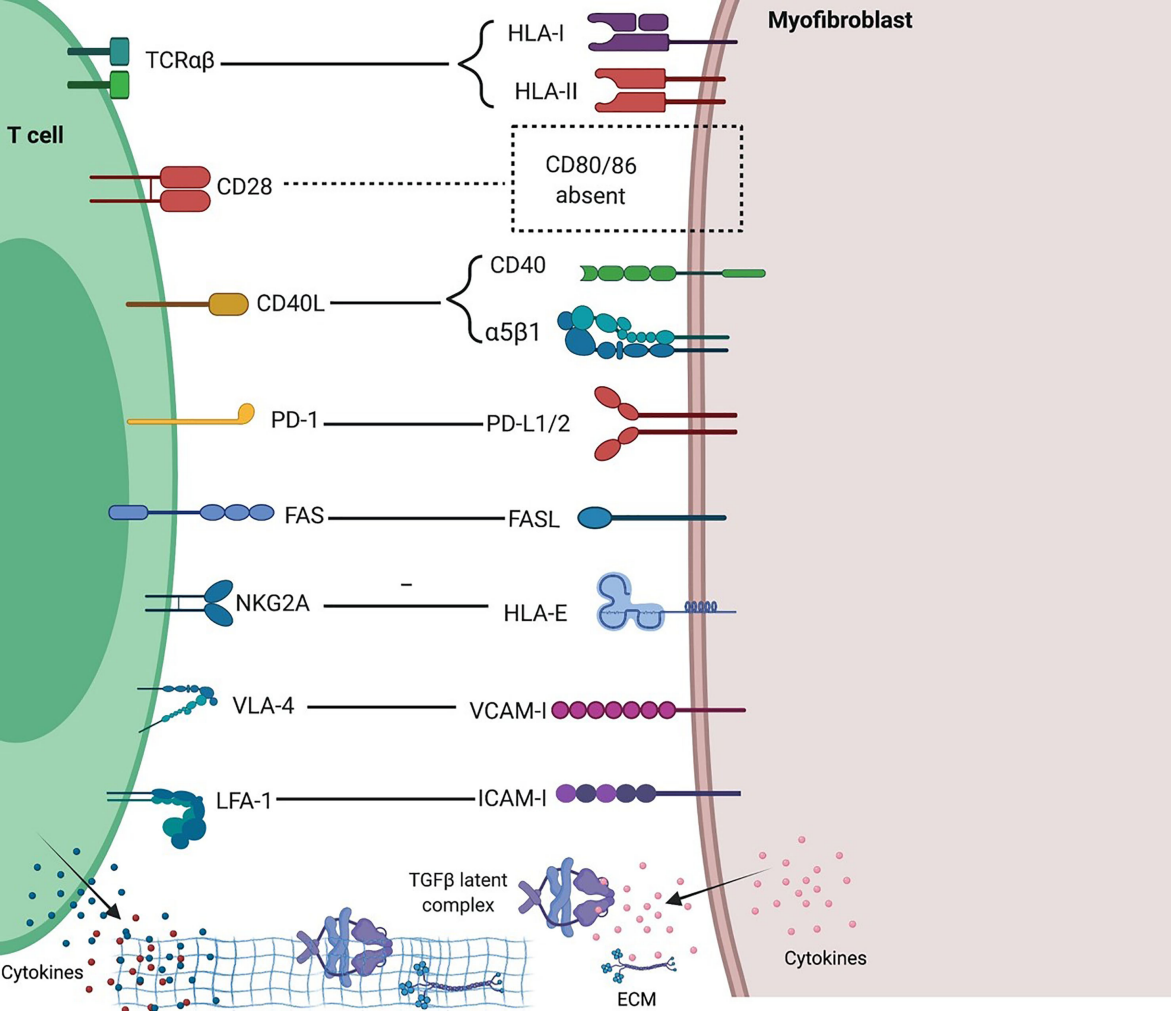
Interactions between T cells and fibroblasts and their implications in fibrosing ILDs. Distinct molecular interactions and reciprocal intracellular signaling exist between heterogenous T cells and fibroblasts in different tissue and immune contexts. Generally, fibroblasts inhibit T cell activation and proliferation due to a lack of potent costimulation and presence of multiple inhibitory signals, and in inflammatory settings such as ILDs, mediate Th1 to Th2 and iTreg to Th17 conversions, thus favoring fibrosis and inflammation. Created with BioRender.com.

Fibroblasts express MHC-I and MHC-II and can cross-present antigens to T cells; however, fibroblasts lack expression of typical costimulatory molecules CD80/86 and IPF T cells exhibit much lower expression of CD28 (81), making IPF fibroblasts unable to activate T cells but rather able to induce T cell anergy or Treg cells (82). IPF fibroblasts acquire an invasive phenotype that is essential for progressive fibrosis, and upregulated PD-L1 on them was required for their invasiveness (8). Furthermore, CAFs in tumors were shown able to kill CD8 T cells in an antigen-specific and antigen-dependent manner *via* PD-L2 and FASL (83). A recent study revealed that senescent fibroblasts expressing HLA-E can evade immune attack by inhibiting cytotoxicity of CD8 T cells and NK cells through HLA-E -NKG2A axis (84) (**Figure 3**). Unlike resident fibroblasts, circulating fibrocytes express CD80^low^ and CD86^high^ as costimulatory molecules, and express PD-L1^high^, but not PD-L2, as a coinhibitory molecule; therefore they strongly enhance the proliferation of CD8 T cells, an effect enhanced by PD-L1 blockade, and have the ability of antigen cross-presentation to CD8 T cells (85). And fibrocytes were also shown able to enhance Th17 response and inflammation (79).

### Paracrine Crosstalk Between Fibroblasts and T Cells

In response to different T cells-derived stimuli, fibroblasts can be immune-activating or suppressing, tissue-destructive or reparative. The roles of different T cell subsets in fibrosis has been reviewed before (86). Th1 cytokines enhance antigen presentation and inflammatory cytokine production, but reduce ECM synthesis in fibroblasts (87). Th2 cytokines IL-4 and IL-13 stimulated upregulation of procollagen and TIMP-1 but downregulation of MMPs, effects partially opposed by Th1 cytokines IFNγ and TNFα (88–91). Likewise, IL-4 is an active player in stimulating collagen synthesis and hyperproliferation of Thy-1^+^ lung fibroblasts, creating a fibrotic environment in the lung (92). IL-4 also enhances expression of adhesion molecules such as beta1 integrin, ICAM-1 and VCAM-1 (93, 94). However, IL-4 secreting Th2 cells from Ssc patients inhibit collagen production by dermal fibroblasts *via* the dominance of membrane-associated TNFα over IL-4 (95). Trans-presentation of cytokines, similar to IL-6 trans-presentation between DCs and T cells (96), occur between fibroblasts and T cells. Fibroblasts produce inflammatory cytokine IL-6 after stimulation with IL-1 or the CD40 pathway (97). Environmental stimulations like irradiation increase expression of IL-6R and gp130 in fibroblasts (98), and IL-6 trans-signaling-STAT3 pathway pe se is able to promote expression of ECM and proliferation markers c-Myc, Bcl-xl and cycline D1 in fibroblasts (99). IL-6 trans-signaling in fibroblasts also induces Tfh (follicular helper T) and B cell differentiation factors responsible for GC formation and fibrosis in the development of IgG4-related disease (100). Treg cells promote lung epithelial proliferation (101) and repair of acute lung injury through production of TGFβ (102) and inhibition of fibrocyte recruitment to the lung along the CXCL12-CXCR4 axis (103).

Fibroblasts are able to orchestrate T cell function and plasticity. Senescent fibroblasts in IPF show abnormal activation, apoptosis resistance, telomere shortening, mitochondrial dysfunction, autophagy deficiency, and senescence-associated secretory phenotypes (SASP) (104). Lung myofibroblasts in bleomycin-induced murine model are sensitive for the activation of SHP2, STAT-3 and SOCS3, but are resistant to IFNγ-STAT-1 activation (105), thus favoring a shift from Th1 to Th2 immune responses. Synovial fibroblasts mediate conversion of iTreg cells to inflammatory Foxp3^-^ Th17 cells by secreting IL-6 (33, 106, 107). Fibroblasts inhibit activation and proliferation of CD4 T cells, inducing a significant reduction of transcription and protein expression of TNFα, CD69, LFA-1 and CD28 in activated CD4 T cells (108). But FLS cells from RA patients induced proliferation of autologous T cells through secreting thrombospondin-1, which recognized CD47 on T cells and induced T cell adhesion, aggregation and costimulation (109). Progressive pulmonary fibrosis is associated with elevated TGFβ production from mechano-stressed AT2 cells during impaired alveolar regeneration (110). Under mechanical stress, integrin-mediated myofibroblast contraction releases active TGFβ from the ECM (111). Constitutive activation of TGFβ2 (but not TGFβ1 or TGFβ3) caused by epigenetic modulation of a discrete TGFβ2 enhancer, which is enforced by BRD4 and NF-κB, drives profibrotic programs in SSc fibroblasts (112). TGFβ1 enhances Fas-mediated lung epithelial cell apoptosis (113), but activates the FAK and the PI3K/Akt anti-apoptotic pathways in lung fibroblasts (114), favoring fibroblast senescence. TGFβ stimulated-human lung fibroblasts secret PD-L1 into extracellular vesicles which are capable of inhibiting T cell proliferation in response to T cell receptor stimulation (115). However, MMPs can cleave membrane-bound PD-L1 on Thy-1^+^ fibroblasts to ablate its suppressive effect on Th1 and Th17 responses (116). PGE2 produced by lung fibroblasts also suppresses apoptosis and activation-induced cell death (AICD) of T cells (117). Fibroblasts also support Th17 expansion through PGE2-mediated upregulation of IL-23 production by DCs (118). IL-11 can be induced by TGFβ (119), and it has been shown to be critical in TGFβ-mediated pulmonary fibrosis (120), which is an ideal target for treatment of ILDs (121, 122). IL-11-expressing fibroblasts and IL-11-ERK signaling in fibroblasts have been demonstrated to play pivotal roles in cancer (123) and Ssc (124). IL-11 polarizes Th17 differentiation through STAT3-RORγt axis in autoimmune diseases (125). However, earlier studies showed that IL-11 polarized Th2 differentiation and inhibited Th1 responses (126), thus preventing acute GVHD. Production of IL-15 by RA synovial fibroblasts induces the proinflammatory cytokines in cocultured T cells which involves IL-15 trans-signaling (127).

Fibroblasts also regulate T cell migration and retention through chemokine gradient and ECM-dependent sequestration of T cells. CD90^+^ adventitial fibroblasts expand and transition to VCAM1^+^ perivascular adventitial fibroblasts in spongiotic dermatitis and lupus which are associated with the retention of dense perivascular T cells (128). Fibroblasts can highly secrete βig-h3, a TGFβ-induced ECM protein, which interacts with CD61 on T cells resulting in phosphorylation of Lck at Y505 and the subsequent inhibition of this early kinase of the TCR signaling pathway (129). In summary, the reciprocal regulation between fibroblasts and T cells is extremely complex in both health and diseases due to the heterogeneity of fibroblasts, T cells and tissues they reside.

## Conclusion

In progressive ILDs, continuously damaged lung tissues are replaced by ECM and myofibroblasts which impede tissue regeneration and lead to gradual decline in lung function. T_RM_ cells and circulating T cells exert adaptive immunity against respiratory infections, as well as inflammation regulation and autoimmunity against self-tissues. The interactions between fibroblasts and T cells in the lung regulate immune responses and tissue remodeling. Understanding thoroughly the interactions between fibroblasts and T cells in the initiation and exacerbation of distinct ILDs, especially IPF, is crucial for developing novel therapies for patients with IPF or other types of ILDs.

Fibroblasts are able to orchestrate T cell function and plasticity. Senescent fibroblasts in IPF are resistant to apoptosis and can also evade immune attack by T cells, favoring fibrosis. Targeting fibroblasts directly has been the mainstay of ILDs therapies, however, specific modulations of T cell function may also indirectly inhibit the profibrotic events of fibroblasts. Harnessing cytotoxicity of T cells against fibroblasts through chimeric antigen receptors has been shown plausible in treating cardiac fibrosis in mice, but remains untested in humans with ILDs. Abrogating TGFβ signaling in TGFBR2-deleted CD4 T cells inhibited tumor growth by reorganizing the reticular vessel system in tumors (130). These CD4 T cells may orchestrate the reshaping of vessel system through interacting with fibroblasts. Therefore, targeting TGFβ signaling in CD4 T cells also holds the potential to treat fibrosing ILDs.

## Author Contributions

YL, XW, and TY contributed equally to this review. All authors contributed to the article and approved the submitted version.

## Funding

This work was supported by Project of The State Key Laboratory of Respiratory Disease (Grant No. SKLRD-Z-202017), grants of the State Key Laboratory of Respiratory Disease, Guangdong-Hong Kong-Macao Joint Laboratory of Respiratory Infectious Disease (Grant No. GHMJLRID-Z-202116 and GHMJLRID-Z-202117), and National Natural Science Foundation of China (Grant No. 82003265).

## Conflict of Interest

The authors declare that the research was conducted in the absence of any commercial or financial relationships that could be construed as a potential conflict of interest.

## Publisher’s Note

All claims expressed in this article are solely those of the authors and do not necessarily represent those of their affiliated organizations, or those of the publisher, the editors and the reviewers. Any product that may be evaluated in this article, or claim that may be made by its manufacturer, is not guaranteed or endorsed by the publisher.
